# Case report: Deep sequencing and long-read genome sequencing refine prior genetic analyses in families with apparent gonadal mosaicism in *PIK3CD*-related activated PI3K delta syndrome

**DOI:** 10.3389/fimmu.2024.1451212

**Published:** 2024-08-26

**Authors:** Halyn Orellana, Jia Yan, Alex Paul, Mari Tokita, Yan Ding, Rajarshi Ghosh, Katie L. Lewis, Joie Davis, Leila Jamal, Colleen Jodarski, Morgan Similuk, Nermina Saucier, Zhanyang Zhu, Yihe Wang, Sitao Wu, Jason Ruggieri, Helen C. Su, Gulbu Uzel, Shareef Nahas, Megan Cooper, Magdalena A. Walkiewicz

**Affiliations:** ^1^ National Institute of Allergy and Infectious Diseases, National Institutes of Health, Bethesda, MD, United States; ^2^ Department of Pediatrics, Division of Rheumatology & Immunology, Washington University School of Medicine in St. Louis, St. Louis, MO, United States; ^3^ Infinity-Biologix LLC (D/B/A SAMPLED), Piscataway, NJ, United States

**Keywords:** gonadal mosaicism, gonosomal mosaicism, *PIK3CD*, inborn errors of immunity, primary immunodeficiency diseases

## Abstract

Gonadal and gonosomal mosaicism describe phenomena in which a seemingly healthy individual carries a genetic variant in a subset of their gonadal tissue or gonadal and somatic tissue(s), respectively, with risk of transmitting the variant to their offspring. In families with one or more affected offspring, occurrence of the same apparently *de novo* variants can be an indicator of mosaicism in either parent. Panel-based deep sequencing has the capacity to detect low-level mosaic variants with coverage exceeding the typical limit of detection provided by current, readily available sequencing techniques. In this study, we report three families with more than one affected offspring with either confirmed or apparent parental gonosomal or gonadal mosaicism for *PIK3CD* pathogenic variants. Data from targeted deep sequencing was suggestive of low-level maternal gonosomal mosaicism in Family 1. Through this approach we did not detect pathogenic variants in *PIK3CD* from parental samples in Family 2 and Family 3. We conclude that mosaicism was likely confined to the maternal gonads in Family 2. Subsequent long-read genome sequencing in Family 3 showed that the paternal chromosome harbored the pathogenic variant in *PIK3CD* in both affected children, consistent with paternal gonadal mosaicism. Detection of parental mosaic variants enables accurate risk assessment, informs reproductive decision-making, and provides helpful context to inform clinical management in families with *PIK3CD* pathogenic variants.

## Introduction

Mosaicism, the presence of multiple genetically distinct cell populations within an individual, is an underrecognized mechanism of pathogenesis in patients with genetic diseases, including inborn errors of immunity (IEI). Parental mosaicism, either gonadal, in which a mosaic variant is present in a subset of gonadal tissue but not somatic tissue, or gonosomal, in which a mosaic variant is present in subsets of both gonadal and somatic tissue, confers risk of transmitting the mutant allele to the offspring(s), which leads to the development of genetic diseases. When unrecognized, parental mosaicism can be mistaken for a *de novo* change and confound recurrence risk estimates. In some clinical situations, e.g., bone marrow donor considerations, it can also influence the clinical management of the affected individual. Nevertheless, testing for mosaicism can be challenging, and current readily available sequencing techniques such as exome with on average 100X depth of coverage, genome with on average 30X depth of coverage, and Sanger sequencing may not provide the depth of coverage needed to detect all clinically relevant mosaic variants. However, in recent years, techniques with higher sequencing depth allowing for a lower limit of detection have proven invaluable in understanding mosaicism in many different areas, particularly for investigating disorders with dominant inheritance that may result from mosaic variants, including IEI.

Specifically, a recent study investigated the incidence of parental gonosomal mosaicism in 92 families with IEIs with *de novo* germline variants and in 36 families with IEIs with moderate-to-high suspicion of gene mosaicism (1). Parental gonosomal mosaicism was detected in approximately 7% of families with *de novo* mutations, and gonadal, somatic, gonosomal, or revertant mosaicism was detected in approximately 64% of families with moderate-to-high suspicion of gene mosaicism (1). Amplicon-based deep sequencing (ADS) showed low-level parental mosaicism in families with IEIs, including activated PI3K delta syndrome 1 (APDS1), autoimmune lymphoproliferative syndrome, Wiskott-Aldrich syndrome, and X-linked agammaglobulinemia (1, 2). This study identified a familial heterozygous c.3061G>A (p.Glu1021Lys) pathogenic variant in *PIK3CD*, defects in which cause APDS1, by Sanger sequencing in two siblings with APDS1. ADS analysis of parental tissue samples in this family, including a paternal sperm sample, did not detect the pathogenic variant in either parent, suggesting maternal gonadal mosaicism in this family (1). Two additional cases of mosaicism for *PIK3CD* have been reported. The first study used next generation sequencing (NGS) based methods on a parent-child trio to elucidate the origin of a homozygous c.3061G>A (p.Glu1021Lys) pathogenic variant in *PIK3CD* in a DNA sample extracted from blood in an affected child. Sanger sequencing did not detect the pathogenic variant in DNA extracted from either parental blood sample. However, ultra-deep sequencing at 2.5 million X depth of coverage of DNA extracted from peripheral leukocytes detected low level mosaicism (1.64% variant allele fraction) for the c.3061G>A (p.Glu1021Lys) *PIK3CD* pathogenic variant in in the apparently healthy mother, consistent with her having gonosomal mosaicism. Further analysis showed segmental uniparental disomy accounting for the presence of the homozygous *PIK3CD* c.3061G>A (p.Glu1021Lys) variant in the child (3). The second study identified a familial heterozygous c.3061G>A (p.Glu1021Lys) pathogenic variant in *PIK3CD* by Sanger sequencing of three half-siblings who shared the same father (4). Sanger sequencing did not detect the pathogenic variant in DNA extracted from paternal or maternal blood samples. However, Sanger sequencing detected the familial *PIK3CD* variant in a paternal DNA sample extracted from semen, which was consistent with paternal gonadal mosaicism, though these results don’t exclude possible low level gonosomal mosaicism.

Delineating parental origin of mosaic findings can inform recurrence risk estimation (5, 6). In families with one or more affected offspring, probabilistic modeling of mosaicism estimates recurrence risk to be higher in cases of maternal gonadal mosaicism compared with cases with paternal origin (5). Further, recurrence risk for additional offspring is estimated to be higher in instances of gonosomal mosaicism compared with mosaicism confined to gonadal tissue for both maternal and paternal transmission (5).

Accordingly, the use of high depth sequencing for detection and diagnosis of low-level mosaicism may be considered for the management and recurrence risk estimation for families with IEIs with multiple affected children and negative parental testing. High coverage NGS-based technologies afford high sensitivity for detecting variants that comprise as little as 1% of the original sample, allowing higher detection of mosaicism. Further, long read sequencing allows phasing of variants when the tissue carrying the variant is unavailable. Here, we present a study demonstrating application of these technologies to elucidate the origin of mosaicism in apparently unaffected parents in three unrelated families with familial heterozygous *PIK3CD* pathogenic variants.

## Case report

**Figure 1:**
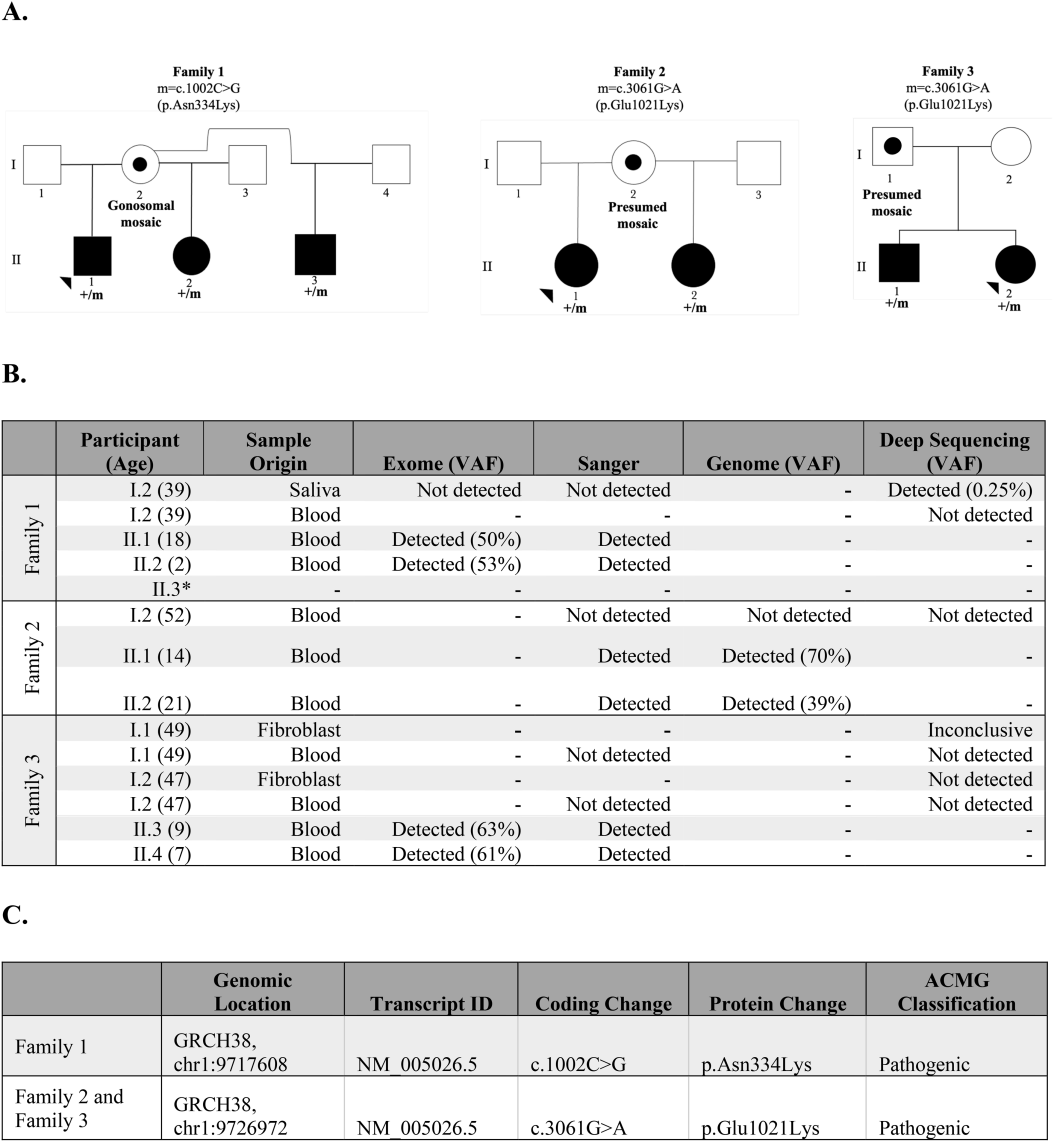
Summary of findings of families 1, 2, and 3. **(A)** Pedigree of the families, including the coding change and the specific amino acid substitution. *Black solid symbols* represent affected subjects, the *open symbol* represents an unaffected subject, the *dotted symbol* represents a subject carrying a postzygotic mutation, *squares* represent male subjects, and *circles* represent female subjects. **(B)** Detection of *PIK3CD* pathogenic variant in exome, Sanger, genome, or targeted deep sequencing in families 1, 2, and 3. **(C)** Genomic information of pathogenic *PIK3CD* variants in families 1, 2, and 3.

In Family 1, we identified a heterozygous c.1002C>G (p.Asn334Lys) pathogenic variant in *PIK3CD* in three half-siblings (II.1, II.2, and II.3) who share the same mother (I.2) (**Figure 1A**). Patient II.1 is an 18-year-old male who showed symptoms of APDS1 starting in infancy, including sinopulmonary infections, splenomegaly, and thrombocytopenia. Half-siblings, patients II.2 and II.3 displayed symptoms from birth; II.2, a 1-year-old female, had cytopenia and leukopenia, while II.3 had cytopenia (**Supplementary Table 1**). Their mother has asthma and allergic rhinitis but is otherwise relatively healthy. A complete list of patient symptoms can be found in **Supplementary Table 1**. Through exome sequencing of genomic DNA samples extracted from whole blood from the proband (II.1) and the half-sibling (II.2), we detected the *PIK3CD* variant in 40/80 reads (50% variant allele fraction (VAF)) and 121/228 reads (53% VAF), respectively (**Figure 1B**). We confirmed both results by Sanger sequencing (**Figure 1B**). The proband’s second half-sibling (II.3) was tested at an outside laboratory and reported to be heterozygous for the familial pathogenic variant. We also performed exome sequencing on a maternal DNA sample extracted from saliva and did not detect the familial *PIK3CD* pathogenic variant at 43 read depth at this locus. We then performed panel-based deep sequencing on the maternal DNA sample extracted from saliva and isolated monocytes. Through this approach, we detected the familial *PIK3CD* variant in 29/11,811 reads (0.25% VAF) in the maternal DNA sample from saliva but did not detect the variant in an additional DNA sample from isolated monocytes. Preliminary analyses of over 500 samples that underwent the same targeted deep sequencing show a mean depth coverage of 8915X at the variant locus in Family 1. For the C>G variant, the mean number of reads across all samples supporting the alternate G allele was 0.42 +/- 1.41, with a maximum of 29 reads, which was significantly above the background. Our results are consistent with low-level gonosomal mosaicism in the mother.

In Family 2, we identified a heterozygous c.3061G>A (p.Glu1021Lys) pathogenic variant in *PIK3CD* in two half-siblings (II.1 and II.2) who share the same mother (I.2) (**Figure 1C**). Patient II.1 and patient II.2 are 14- and 21-year-old females, respectively, diagnosed with APDS1 and developed symptoms of APDS1 during infancy, starting with otitis media (**Supplementary Table 1**).Their mother has Crohn’s disease. Through genome sequencing of genomic DNA extracted from whole blood from the proband (II.1) and half-sibling (II.2), we detected the *PIK3CD* variant in 21/30 reads (70% VAF) and 14/36 (39% VAF), respectively (**Figure 1B**). We confirmed both results by Sanger sequencing. We also performed genome sequencing on a maternal DNA sample extracted from blood and did not detect the familial *PIK3CD* pathogenic variant. We performed deep sequencing at an average depth of 9,677 X coverage at the *PIK3CD* locus to detect possible low-level mosaicism in her blood sample. Through this approach, we did not detect the c.3061G>A (p.Glu1021Lys) pathogenic variant. This finding suggests the presence of gonadal mosaicism in the proband’s mother, which could not be experimentally confirmed because of the non-availability of gonadal tissue for testing. However, we cannot rule out the highly low probability of recurrence of a *de novo* variant.

**Figure 2:**
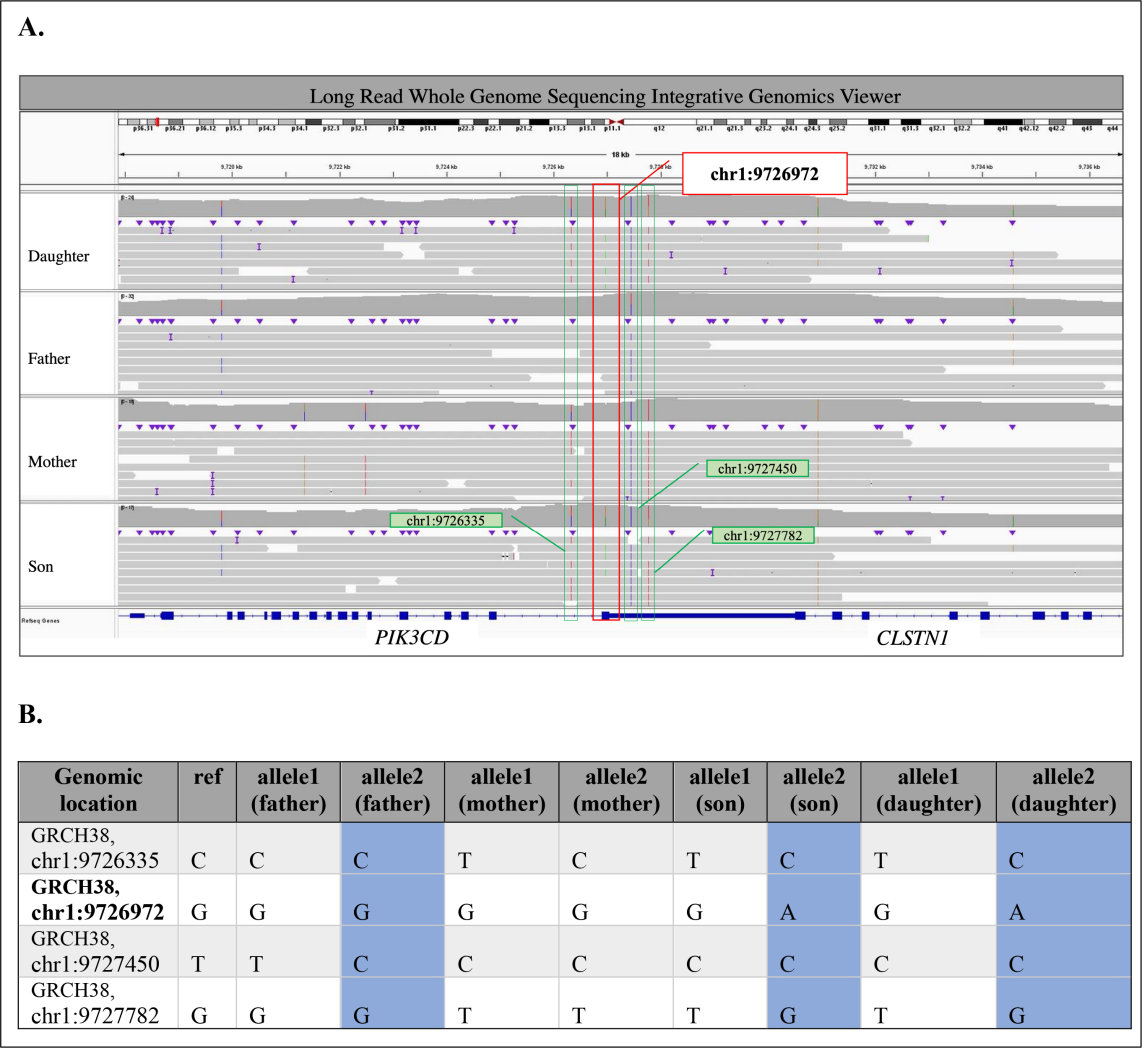
Summary of Long Read Whole Genome Sequencing analysis in Family 3. **(A)** Variants near the pathogenic c.3061G>A (p.Glu1021Lys) *PIK3CD* variant (chr1:9726972G>A; highlighted in red box) in Integrative Genomics Viewer (https://www.igv.org) for Daughter, Father, Mother, and Son from top to bottom panel. Variants surrounding pathogenic variant are highlighted in green boxes. **(B)** Phased variants near the pathogenic c.3061G>A (p.Glu1021Lys) *PIK3CD* variant (chr1:9726972G>A; in bold). Shared paternal haplotype is highlighted in blue.

In Family 3, we identified a familial heterozygous c.3061G>A (p.Glu1021Lys) pathogenic variant in *PIK3CD* in two affected siblings (II.1 and II.2) born to non-consanguineous parents (I.1 and I.2) of reportedly diverse genomic ancestry (**Figure 1C**). Patient II.1 is a 9-year-old male who showed symptoms of APDS1 starting at age four, primarily sinopulmonary infections. Patient II.2 is a 7-year-old female that displayed symptoms of APDS1 at one year of age, initially presenting with thrombocytopenia (**Supplementary Table 1**). Their mother and father are both apparently unaffected with no major health issues. Through exome sequencing of genomic DNA extracted from whole blood from the proband (II.1) and the sibling (II.2), we detected the *PIK3CD* variant in 27/43 reads (63% VAF) and 19/31 reads (61% VAF), respectively (**Figure 1B**). We confirmed these results through Sanger sequencing. We also performed Sanger sequencing on the parental DNA samples extracted from whole blood and we did not detect the familial pathogenic variant in either sample. Additionally, previously completed exome sequencing at an outside lab did not detect the variant in either parent. We performed deep sequencing on parental genomic DNA samples extracted from both blood and fibroblasts. However, at an average depth of 9,677 X coverage at the *PIK3CD* locus, we did not detect the familial pathogenic variant in blood-derived DNA samples from either parent. Deep sequencing of the DNA extracted from fibroblast samples did not detect the variant in the mother and results for the father were inconclusive. We performed long-read genome sequencing on DNA samples extracted from blood from all four members of Family 3 to determine the parental chromosome of origin harboring the c.3061G>A (p.Glu1021Lys) pathogenic variant in the two affected siblings. Haplotype analyses of chromosomal regions surrounding the pathogenic allele showed that the pathogenic variant in *PIK3CD* resides on the paternal allele in both affected children (**Figure 2**) (**Supplementary Methods**). Together, these findings are best explained by gonadal mosaicism in the father.

## Discussion

The presence of gonadal or gonosomal mosaicism is strongly suggested when a dominantly inherited disease recurs in offspring of apparently healthy parents; such cases have been reported in families with *PIK3CD* pathogenic variants (1, 3, 4). Here, we report a case series of three families with either confirmed or apparent gonosomal or gonadal mosaicism for pathogenic *PIK3CD* variants. These families had multiple affected children with the same pathogenic *PIK3CD* variant. Parents in each family were unaffected and did not have any apparent symptoms attributed to APDS1. Data from targeted deep sequencing following exome and Sanger sequencing supported maternal gonosomal mosaicism in Family 1. Through this approach we did not detect the pathogenic variant in maternal blood samples for Family 2, suggesting that mosaicism was likely confined to her gonads. Data from long-read genome sequencing following inconclusive targeted deep sequencing supported paternal gonadal mosaicism in Family 3. Although, we did not measure the level of mosaicism in the gametes, we hypothesize that the level of mosaicism is likely higher in the gonads than what we observed in peripheral tissues, based on our findings in these three families. Additional studies are needed to determine whether higher mosaicism in the gonads could account for the 100% incidence of affected children in each family we examined.

Our data from Families 1 and 2 are consistent with theoretical calculations of increased germline mosaicism in maternal samples (5). In families with more than one affected child and apparently unaffected parents, the likelihood of maternal mosaicism for the *PIK3CD* variant is increased (1, 3). Furthermore, data from Family 1 supports theoretical increased recurrence risk estimates due to the presence of maternal gonosomal mosaicism, as gonosomal mosaicism and maternal origin are both major determinants of elevated recurrence risk compared with gonadal mosaicism and paternal origin, respectively. (5). This is because an apparently mosaic parent with multiple affected offspring may have a larger fraction of variants in their germline cells than is typical, making transmission of a mutant gamete more likely.

In Family 3, deep sequencing data could not conclusively identify the parental origin of the pathogenic *PIK3CD* variant in the affected siblings. Long-read sequencing on parental blood samples with haplotype analysis was used to delineate the parent of origin of the transmitted allele in the offspring (7). By phasing the surrounding variant regions in the affected siblings with the parental chromosome that had shared single nucleotide variants (SNVs) we determined which parent transmitted the risk haplotype. We captured unique SNVs from each parent, as they were each from diverse genomic ancestries. This analysis demonstrated the utility of long-read sequencing to investigate parent of origin following inconclusive deep sequencing.

Detection of mosaicism has serious implications in hematopoietic stem cell transplantation for families with affected offspring and potential donor parents; however, the results of this study do not impact the clinical management of the affected patients themselves. Detection of low-level mosaicism through targeted deep sequencing of a parent prior to transplantation could reduce the likelihood of bone marrow rejection or failure, as mosaic parents are not suitable donors due to potential hematopoietic phenotypes conferred by the mosaic variant.

Additionally, clarifying that an apparently *de novo* variant is actually due to parental gonadal or gonosomal mosaicism is important for providing accurate recurrence risk estimates. Genetic counselors often rely on an empirical understanding of recurrence risk. Elucidating low level parental mosaicism in families with affected children with apparently *de novo* variants can provide families with more accurate recurrence risk assessment during counseling sessions. Recurrence calculations can help guide family planning discussions and direct the use of resources such as preimplantation and prenatal genetic diagnostic testing (8).

Although prior studies showed mosaicism that was stable over time in families with IEIs (1, 9), individuals with mosaicism may show later disease onset and/or milder or subclinical phenotypes than individuals with non-mosaic variants in the same genes (10). The level of mosaicism in *PIK3CD* at which APDS1 develops is unknown at this time and it may depend on the variant type and the cells types in which it is expressed. A prior report of a mother with gonosomal mosaicism for *PIK3CD* at 37 years of age with1.64% VAF had no apparent clinical phenotype (3). Similarly, the mother we identified in Family 1 at the time of evaluation at 39 years of age, also had no apparent clinical phenotype with 0.25% VAF. At this time, we have no data to determine her clinical course in the future. Based on prior publications suggesting stability of *PIK3CD* mosaicism over time, we hypothesize that that the level of mosaicism will remain stable and not lead to subsequent phenotypic manifestation of disease in the unaffected parents; nevertheless, this hypothesis lacks experimental confirmation (1, 3). Given our limited understanding of the dynamics of mosaicism and subsequent disease progression, future studies characterizing its cellular and clinical spectrum throughout life are warranted.

This report should be interpreted in light of several limitations. First, the limited availability of tissues for testing did not allow for a wide view of the landscape of mosaicism across multiple cell lineages in these families. Further, the depth of deep sequencing of 9,677X may limit detection of lower levels of mosaicism than what we have detected in this study. Finally, data on the positive and negative predictive values for deep sequencing require additional longitudinal studies that follow phenotypic features of participants with varying VAFs. The extent of baseline mosaicism of IEI genes in healthy individuals across the lifespan and the clinical impact of mosaic changes remains to be addressed.

In summary, as we describe in this study, detection of mosaic variants in *PIK3CD* in seemingly healthy parents improves risk assessment and clinical management for families with apparently *de novo* variants in more than one offspring.

## Data Availability

The datasets presented in this study can be found in online repositories. The names of the repository/repositories and accession number(s) can be found below: dbGaP accession ID phs001899.v3.p1.

## NIAID Centralized Sequencing Program Working Group

The members of the NIAID Centralized Sequencing Program working group are Bryce A. Seifert, Nadjalisse Reynolds-Lallement, Rachel Gore Moses, Michael Kamen, Sarah Bannon, Sophie Byers, Ekaterina Damskey, Sruthi Srinivasan, Adrienne Borges, and Nicole Gentile.
