# Supplementary material for: Case report: Deep sequencing and long-read genome sequencing refine prior genetic analyses in families with apparent gonadal mosaicism in PIK3CD-related activated PI3K delta syndrome
Source: Front Immunol. 2024 Aug 26;15:1451212. doi: 10.3389/fimmu.2024.1451212 (PMC11381395; doi:10.3389/fimmu.2024.1451212)
Supplement: Supplementary file 1 [file DataSheet1.pdf]

Supplementary Materials

Supplemental Table 1. Participant phenotypes.

	Participant	Primary diagnosis	Phenotypes
Family 1	I.2		Allergic rhinitis, asthma, astigmatism, blood dyscrasia, depression, eczema, myopia
	II.1	Activated PI3K-delta syndrome	Asthma, autoimmune thrombocytopenia, splenomegaly, bronchiectasis, cholelithiasis, chronic lung disease, decreased circulating antibody level, fever, headache, hypotension, immunodeficiency, lymphadenopathy, lymphoid nodular hyperplasia, meningitis, osteopenia, persistent CMV viremia, persistent EBV viremia, pneumonia, portal hypertension, recurrent candida infections, recurrent shingles, recurrent sinopulmonary infections, seizure, sepsis, tachycardia
	II.2	Activated PI3K-delta syndrome	Combined immunodeficiency, cytopenia, leukopenia, lymphadenopathy, recurrent infections, enteropathy, eosinophilic esophagitis, acute bilateral conjunctivitis, autism spectrum disorder, global developmental delay, incontinence of feces, incontinence of urine, macrocephaly, picky eater, respiratory infection, hyperopia of both eyes
	II.3	Activated PI3K-delta syndrome	Cytopenia
Family 2	I.2	Crohn's disease	Hypertension, hypothyroidism
	II.1	Activated PI3K-delta syndrome	Recurrent otitis media, allergy, asthma, bronchiectasis, delayed gross motor development, delayed speech and language development, exocrine pancreatic insufficiency, hypothyroidism, immunodeficiency, lactose intolerance
	II.2	Activated PI3K-delta syndrome, Common Variable Immunodeficiency	Abnormality of ethmoid sinus, asthma, bronchiectasis, carious teeth, chronic sinusitis, dysphagia, global developmental delay, hearing impairment, irregular menstruation, moderate conductive hearing impairment, recurrent otitis media, sinusitis
Family 3	I.1		
	I.2		
	II.1	Activated PI3K-delta syndrome	Sinopulmonary infections, headache, hypothyroidism, left atrial enlargement, precocious puberty, recurrent otitis media, recurrent sinusitis, rhinorrhea, stomatitis, syncope, verrucae
	II.2	Activated PI3K-delta syndrome	Cough, hypothyroidism, meningitis, osteomyelitis, pulmonary infiltrates, recurrent otitis media, recurrent pneumonia, short stature, sinusitis, splenomegaly, thrombocytopenia

Supplemental Methods

Exome Sequencing

Detailed methods for exome sequencing can be found in Similuk et al. (1). Briefly, exome sequencing was performed on the Illumina HiSeq2500 sequencing system for Family 3 and on the Illumina NovaSeq 6000 instrument for Family 1 with a minimum coverage of 95% > 20X and mean coverage of 100X for identification of variants related to the clinical presentation of affected subjects (1). Relevant variants were confirmed by Sanger sequencing or other appropriate methods meeting Clinical Laboratory Improvement Amendments/ College of American Pathologist (CLIA/CAP) requirements. Confirmation of genomic sex and relationship was based on estimates of identity by descent.

Genome Sequencing

Research-based genome sequencing was performed on an Illumina sequencing system with a minimum coverage of 95% > 20X and mean coverage of 30X for targeted genomic regions (2). Relevant variants were confirmed by Sanger sequencing meeting CLIA/CAP requirements.

Targeted Deep Sequencing

Next generation sequencing-based targeted deep sequencing was performed for the detection of low-level mosaic variants in *PIK3CD* with an average coverage of 9,677X.

Pacbio Long Read Whole Genome Sequencing (LR-WGS)

Long Read Whole Genome Sequencing (LR-WGS) was performed on quad samples (2 siblings and 2 parents) using Pacbio Single Molecule Real-Time (SMRT) chemistry and Revio sequencing platform that consisted of two processing steps: First, library construction and second SMRT sequencing.

Library construction was performed using between 3 to 5 ug of high molecular weight (50% ≥30 kb & 90% ≥10 kb) gDNA in non EDTA buffer following manufacturer protocol according to Similuk et al. (1). The gDNA was sheared to generate fragments ranging from 15 to 25Kb. The fragmented gDNA went through multiple library construction steps including DNA damage Pacbio repair, End-repair/A-tailing, adapter ligation, beads clean up and size selection using Pacbio HiFi SMRTbell Library Prep Kit 3.0 (catalog #: 102-182-700). Library QC to ensure fragment sizes were between 15-25 Kb was performed using Agilent fragment analyzer. Library concentrations were determined using Qubit to ensure between 20-60 ng/μL prior to sequencing. The final libraries were prepped for sequencing according to instructions from Pacbio SMRT Link v13.0 (a software interface to allow a user to interact with for loading a SMRTbell library and enabling downstream streamlined analysis workflows), at a recommended on-plate-loading-concentration between 200-300 pM. The library was then dispensed into a Revio sequencing plate (catalog #: 102-587-400) and loaded on the Revio system along with a SMRTcell tray (catalog #: 102-587-400) for sequencing targeting a depth of 20x. At least 70 Gb HiFi reads per sample were generated for downstream analysis. Sequencing data was generated with PacBio Circular consensus sequencing (CCS) analysis for each of the four samples using Pacbio SMRT Link 13.0.

Bioinformatics data analysis for each set of LR-WGS raw sequencing data was performed in the following steps as shown in figure 1. First, with raw unaligned PacBio BAM files as input, PBMM2 software (version 1.13.1, https://github.com/PacificBiosciences/pbmm2) was used to align long reads against human genome (GRCh38) to generate aligned BAM files. This was followed by running Deep Variant software version 1.5.0 (3) to call small variants and genomic variants were saved in VCF files. Finally, phased genomic variants were generated using the software WhatsHap software version 2.0 (4). Genome coverages were calculated by using Mosdepth version 0.3.4 (5) as shown in Table 1 for four samples. SNP genotypes near the variant of interest presented in both sibling samples "chr1:9726972G>A" are shown in Figure 2A using IGV (Integrative Genomics Viewer). For the phased VCF files, four phased variants around "chr1:9726972G>A" are listed in Figure 2B. From both Figure 2A and Figure 2B, variant "chr1:9726972G>A" was determined to be inherited from father (blue/grey color from father/mother in Figure 2B).

Figure 1. Flowchart of analysis pipeline

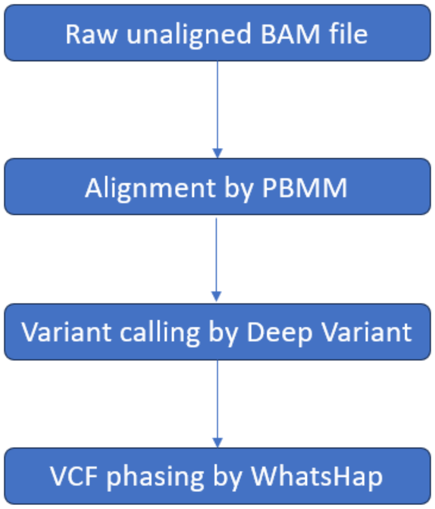

Table 1. Genome coverage for 4 samples

Sample	Genome coverage (fold X)
Mother	31.24
Daughter	22.75
Father	28.95
Son	22.92

References

1. Similuk MN, Yan J, Ghosh R, Oler AJ, Franco LM, Setzer MR, et al. Clinical exome sequencing of 1000 families with complex immune phenotypes: Toward comprehensive genomic evaluations. J Allergy Clin Immunol. 2022 Oct 1;150(4):947–54.

2. Ghosh R, Bosticardo M, Singh S, Similuk M, Delmonte OM, Pala F, et al. FOXI3 haploinsufficiency contributes to low T-cell receptor excision circles and T-cell lymphopenia. J Allergy Clin Immunol. 2022 Dec;150(6):1556–62.

3. Poplin R, Chang PC, Alexander D, Schwartz S, Colthurst T, Ku A, et al. A universal SNP and small-indel variant caller using deep neural networks. Nat Biotechnol. 2018 Nov;36(10):983–7.

4. Martin M, Patterson M, Garg S, Fischer SO, Pisanti N, Klau GW, et al. WhatsHap: fast and accurate read-based phasing [Internet]. bioRxiv; 2016 [cited 2024 Mar 7]. p. 085050. Available from: https://www.biorxiv.org/content/10.1101/085050v2

5. Pedersen BS, Quinlan AR. Mosdepth: quick coverage calculation for genomes and exomes. Bioinformatics. 2018 Mar 1;34(5):867–8.
